# Catheter Closure of Clinically Silent Patent Ductus Arteriosus Using the Amplatzer Duct Occluder II-Additional Size: A Single-Center Experience

**DOI:** 10.7759/cureus.17481

**Published:** 2021-08-27

**Authors:** Yasser A Bhat, Abdulrahman Almesned, Abdullah Alqwaee, Ali Al Akhfash

**Affiliations:** 1 Department of Pediatric Cardiology, Prince Sultan Cardiac Centre, Buraidah, SAU

**Keywords:** patent ductus arteriosus closure, amplatzer occluder, infective endocarditis, transcatheter closure device, arterial injury

## Abstract

Objectives

Transcatheter closure is the treatment of choice for most patent ductus arteriosus (PDA) in infants, children, and adults. However, there is a controversy regarding transcatheter closure of clinically silent PDAs. Some authors favor device closure to eliminate the lifelong risk of infective endarteritis while others recommend avoiding PDA closure in such patients. The study describes our experience of closing the silent PDAs using the Amplatzer duct occluder II-additional size (ADO II-AS) (St. Jude Medical Corp, St. Paul, MN).

Materials and methods

From April 2018 through March 2021, 52 consecutive pediatric patients aged 18 years and less with clinically silent PDA who had transcatheter closure at our center were enrolled. Patients were excluded if they had clinically detected PDAs; had surgical ligation of PDA with no residual shunt; had left heart dilatation on echocardiography; or moderate-sized PDAs closed with ADO II-AS. In addition, patients with an innocent murmur or murmur due to an associated lesion were included. This study was retrospective, and all of the 52 patients underwent PDA device closure using ADO II-AS.

Results

Fifty-two consecutive patients were enrolled with a median age of 17 months, range (97-2.5) 94.5 months. Mean weight was 11.29 kilogram, range (24.8-3.5) 21.3 kilogram, and mean follow-up was 13.5 months, range (29-0) 29 months. Thirty-one (59.6%) were females, and 21 (40.4%) were males. The mean procedure time was 30.6 min, range (60-10) 50 min, and mean fluoroscopic time was 5.5 min, range (28-1.7) 26.3 min. The mean volume of contrast given was 9.1 milliliter, range (30-4) 26 milliliter. Forty-five (45; 88.2%) patients had immediate closure of PDA. No patients had anesthetic or vascular complications; however, two patients had procedural complications. Device placement was unsuccessful in one patient with Downs syndrome. The mean follow-up for our patients was 13.5 months, range (29-0) 29 months; the patients were asymptomatic at the follow-up, and none of the patients had any residual leak. None of the patients showed coarctation or left pulmonary artery stenosis at the latest follow-up.

Conclusion

The usefulness of catheter-based therapy for silent PDA is less well-established by current evidence. Further studies are needed to justify the intervention solely based on the premise that the silent duct is a substrate for infective endarteritis; however, our reason to close silent PDA was to do so primarily because of social reasons. This study found that device closure of silent PDA is safe and effective using an ADO II-AS device with minimal risk of embolization and a low residual shunt rate. Coils have been used to close small PDAs, however, with higher rates of embolization and device malpositioning. We believe ADO-II AS offers an advantage of safety and efficacy over coils. In addition, the study highlights the advantage of using an ADO II-AS device, which can be delivered via a four French delivery system with no arterial complications.

## Introduction

Transcatheter closure is the treatment of choice for most patent ductus arteriosus (PDA) in infants, children, and adults [1], but there is a controversy regarding transcatheter closure of clinically silent PDA. Some authors favor device closure of silent PDA to eliminate the lifelong risk of infective endarteritis [2-3]. They claim that experience is growing with device-based therapy, and there is a decrease in morbidity and mortality with catheter intervention. As such, the consequences of infective endocarditis make the risk of an intervention an acceptable alternative [2].

Furthermore, the risk of endocarditis may not be related to the size of the duct or the presence of a continuous murmur, and all the patients with unexplained bacteremia should be evaluated for silent PDA [3]. In contrast, others do not favor routine closure of silent PDA and argue that the extremely low risk of infective endarteritis doesn't justify the closure of silent duct at the expense of morbidity, mortality, and cost associated with the procedure [4-6]. The study describes our experience of closing silent PDAs using the Amplatzer duct occluder II-additional size (ADO II-AS) (St. Jude Medical Corp, St. Paul, MN). Amplatzer duct occluder II-additional size is a fabric-free device made of nitinol wire mesh designed to close PDA with the small aorta and left pulmonary artery. It can be delivered via a small catheter using an antegrade or retrograde approach [7].

## Materials and methods

Between April 2018 and March 2021, 52 consecutive pediatric patients aged 18 years and less with clinically silent PDA who had a transcatheter closure at our center participated in the study. The institutional review board of Prince Sultan Cardiac Centre, Qassim, approved the study (111831) and granted an informed consent waiver. Patients were excluded if they had clinically detected PDAs (presence of continuous murmur) closed by the transcatheter method; had surgical ligation of PDA but no residual shunt; had left heart dilatation on echocardiography; had moderate-size PDAs closed with ADO II-AS. However, patients with an innocent murmur or murmur due to an associated lesion and patients having residual PDA after surgical ligation of the duct were included. The study was retrospective, and all of the 52 patients underwent PDA device closure using ADO II-AS. We reviewed pre-procedure and post-procedure records of all patients up to the most recent follow-up, including the echocardiographic images, catheterization reports, and discharge summaries. Since 2018, our health institution has had an electronic health record system, and all the records are stored electronically. Data regarding patient characteristics, echocardiographic findings, procedure details, outcomes, and adverse events were collected and recorded for analysis. Clinically silent PDA was defined as one that is not detected clinically (no continuous murmur on auscultation) but visualized on color flow echocardiography [6].

Procedure details

An aortogram was performed using a multipurpose catheter (MPA2) in anteroposterior and lateral projections to delineate PDA anatomy. After measuring the PDA length and the narrowest diameter, device size was chosen based on the discretion of the interventionist. The device was delivered via the Amplatzer^TM^ TorqVue^TM^ Low Profile Delivery System (AGA Medical Corporation, North Plymouth, MN). The PDA was crossed with a guidewire, and the Torq Vue delivery sheath was advanced over the wire across the PDA. The pre-attached ADO II-AS device to the delivery cable was advanced to the tip of the delivery sheath. Once the first disc was released by advancing the delivery cable slowly, the whole assembly of the delivery sheath, cable, and device was then withdrawn to bring the released disc in opposition to the PDA wall using the tracheal shadow as a landmark on lateral angiography. Next, the device was deployed by withdrawing the delivery sheath over the delivery cable. Finally, angiography was performed through the side port of the delivery sheath to check the device position, and when satisfied with device positioning, the device was unscrewed and released. After the device deployment, an aortogram was performed to assess the arch, check the device position, and see any residual shunt. Aortic and pulmonary artery pressures were not routinely measured after device placement.

## Results

Patient characteristics

Fifty-two consecutive patients were enrolled with a median age of 17 months, range (97-2.5) 94.5 months. The mean weight was 11.29 kilograms, range (24.8-3.5) 21.3 kilograms. Four (7.7%) patients had pulmonary valvuloplasty as the first intervention, followed by PDA device closure in the same sitting. All the patient characteristics, procedure details, and outcomes are summarized in Table 1.

**Table 1:** Showing indications for cardiac evaluation for patients undergoing device closure for silent PDA *Data was not available for 22 patients. PDA: patent ductus arteriosus

Indication for cardiac evaluation	Number of patients
Murmur due to an associated lesion	Severe pulmonary stenosis, 4
	Moderate aortic stenosis, 1
	Small VSD, 3
	(15.3) percent
Downs syndrome	6 (11.5) percent
Prematurity	4 (7.6) percent
Innocent murmur	3 (5.7) percent
Other siblings having congenital heart disease	2 (3.8) percent
Referred from other centers as tiny PDA	2 (3.8) percent
Other unspecified syndromes	2 (3.8) percent
Infant of diabetic mother	1 (1.9) percent
Cardiomegaly on chest X-ray	1 (1.9) percent
Staus post ventricular septal defect surgical closure (Had Downs syndrome)	1 (1.9) percent
Status post-coarctation repair in the newborn period with residual PDA	1 (1.9) percent
Post coarctation balloon angioplasty	1 (1.9) percent
Deficient data*	22 (42.3) percent

Procedural outcomes

The majority of the procedures were performed under conscious sedation, but patients with difficult airways, such as those with Down syndrome and small babies who have had a concomitant balloon pulmonary valvuloplasty, have been given general anesthesia. The four French delivery system was utilized in all the retrograde approach procedures, whereas the five French delivery sheath was utilized for the antegrade route. Patients did not have hemodynamic assessment routinely except in some selected situations.

Four patients with severe pulmonary stenosis had a right heart hemodynamic assessment. One patient who had a mild coarctation underwent hemodynamic evaluation before and after device placement; however, there was no increase in gradient between the ascending and descending aorta after the device deployment. Moreover, another patient who had coarctation balloon angioplasty in infancy demonstrated no coarctation before and after device placement. Although two patients with Downs syndrome had hemodynamic assessment before device placement, it revealed normal pulmonary artery pressures. Both of these patients had pulmonary hypertension secondary to non-cardiac causes, which had improved over time.

Device placement was unsuccessful in a seven-year-old child with Downs syndrome with a tiny PDA. After crossing the PDA with a guidewire, the delivery sheath could not be negotiated across the PDA; therefore, the procedure was abandoned. In the follow-up, this patient had spontaneous closure of the PDA. No patients had anesthetic or vascular complications; however, two patients had procedural complications.

Although a one-year-old female developed dissection of the PDA while advancing the delivery sheath over the guidewire across the PDA, it was not clinically significant. The patient was last evaluated six months after the procedure and was doing well with no sequelae. In another patient, a one-year-old female, even though the device was embolized to the right pulmonary artery after release, it was successfully captured by the snare and retrieved, and a larger device was implanted in the same sitting.

One patient had Kawasaki disease with left anterior descending artery ectasia; he had a small PDA, which was successfully closed. Another patient had undergone ventricular septal defect closure and PDA ligation in early infancy; he had a small residual PDA closed by the device. Predischarge echocardiography was performed on the same day of the intervention. One (1.9%) patient had a residual leak, and no patient had left artery stenosis or coarctation.

The mean follow-up for our patients was 13.5 months, range (29-0) 29 months. All the patients were asymptomatic at follow-up, and none of the patients had any residual leak. None of the patients showed coarctation or left pulmonary artery stenosis at the latest follow-up. Table 2 shows the characteristics, procedure details, and outcomes of patients.

**Table 2:** Showing the patient characteristics, procedure details, and outcomes *Data was available for 50 patients only PDA: patent ductus arteriosus; BSA: body surface area; BMI: body mass index; ADO II-AS: Amplatzer duct occluder II-additional size

Variable	Statistical value
Age (months), median	17
Weight (kg), mean	11.3
Height (cm), mean	82.2
BSA (m^2^), mean	0.48
BMI (kg/m^2^), mean	16.03
Sex	Females: 31 (59.6%)
	Males: 21 (40.4%)
Non-syndromic vs syndromic	Non-syndromic: 42 (82.7%)
	Downs syndrome: 7 (13.5%)
	Others: 2 (3.8%)
Concomitant interventional procedure	Balloon pulmonary valvuloplasty: 4 (7.7%)
Type of anesthesia	Conscious sedation: 42 (80.8%)
	General: 10 (19.2%)
Approach	Retrograde: 47 (90.4%)
	Antegrade: 5 (9.6%)
Access sheath size (French)	4 French: 48 (92.3%)
	5 French: 4 (7.6%)
Hemodynamic assessment	No, 44 (84.6%)
	Yes, 8 (15.4%)
Device size (ADO II-AS) in mm	4 x 4 mm, 13 (25.4%)
	5 x 4 mm, 10 (19.6%)
	5 x 2 mm, 9 (17.6%)
	4 x 6 mm, 7 (13.7%)
	5 x 6 mm, 6 (11.7%)
	Others, 6 (11.7%)
Procedure time in min^*^, mean	30.6
Fluoroscopic time in min^*^, mean	5.5
Contrast given in millilitre^*^, mean	9.1
Residual PDA after device deployment(immediate efficacy)	45 (88.2%)
	6 (11.8%)
Anesthetic complications	0 (0%)
Procedural complications	2 (3.8%)
Vascular complications	0 (0%)
Predischarge echocardiographic findings	Residual PDA: 1 (1.9%)
	Left pulmonary artery stenosis: 0 (0%)
	Coarctation of aorta: 0 (0%)
Follow-up in months, mean	13.5
Symptoms at latest follow-up	0 (0%)
Echocardiographic findings at the most recent follow-up	Residual PDA: 0 (0%)
	Left pulmonary artery stenosis: 0 (0%)
	Coarctation of aorta: 0 (0%)

## Discussion

The management of the silent PDA is a dilemma for the pediatric cardiologist [8]. Houston and colleagues described silent PDA in patients with a continuous spectral Doppler pattern on echocardiography without continuous or crescendo murmur and no evidence of pulmonary hypertension. Authors in this study described 20 children with silent PDA and suggested that infective endocarditis in a normal heart can have a tiny PDA as a predisposing factor; however, they don't advise routine closure of silent PDA [9]. Thilén et al. reviewed the death certificates of 270 pediatric and adult people having infective endarteritis with a PDA. Out of 30-million deaths between 1960 and 1993, only two cases were due to infective endarteritis as a PDA complication. Therefore, they don't recommend the routine closure of silent PDA [10].

Another study reported infective endarteritis as a complication of PDA in 14 children over six years; most of these patients had small PDAs [11]. PDA-associated endarteritis is rare but can occur when the duct is still open or as it closes [12]. Patrícia Sá Ferreira et al. reported an infant with Kleibsella endarteritis concerning a silent PDA [13]. A scientific statement from the American Heart Association recommends considering transcatheter closure of silent PDA [14].

Since 2018, we have had an institutional policy of closing all silent PDAs. Apart from the risk of infective endarteritis, this approach was mainly adopted because of social reasons. There is a stigma attached to congenital heart disease in a developing country like ours, whether complex or straightforward, especially for females who may have difficulty seeking a match.

ADO II-AS was used in all our study patients. The ADO II-AS consists of a single layer of nitinol wire braid, replacing the polyester fabric of ADO. It provides a lower profile for device delivery (4 French for all PDA sizes) [7]. In addition, ADO II-AS has a central waist and two retention discs, which are only one mm larger than the waist. The low-profile retention discs and end screw design minimize the protrusion of the device into the aorta or left pulmonary artery [15]. We primarily selected ADO II-AS for use in our study group because of its low profile to avoid vascular complications and smaller retention discs to minimize the risk of protrusion of the device into the aorta and left pulmonary artery. In this study, none of the patients had any vascular complications, coarctation of the aorta, or left artery stenosis after PDA device closure.

PDA coil closure needs a larger catheter for deployment and has longer fluoroscopic times [16]; it has higher embolization rates and device malpositioning [17]. This study used four French delivery systems in most patients with a mean fluoroscopic time of 5.5 min. We don't use cine-angiography in the catheterization laboratory to minimize radiation exposure to patients and catheterization laboratory staff. Although the device was embolized to the right pulmonary artery in one patient, it was successfully captured and retrieved without any adverse events. The device embolized may be due to the selection of a smaller size device. At a mean follow-up of 13.5 months, none of our patients had any complications or residual shunt.

## Conclusions

The usefulness of catheter-based therapy for silent PDA is less well-established by current evidence. Further studies are needed to justify the intervention solely based on the premise that the silent duct is a substrate for infective endarteritis; however, our reason to close silent PDA was to do so primarily because of social reasons. This study found that device closure of silent PDA is safe and effective using an ADO II-AS device with minimal risk of embolization and low residual shunt rate. Coils have been used to close small PDAs, however, with higher rates of embolization and device malpositioning. We believe ADO-II AS offers the advantages of safety and efficacy over coils. In addition, the study highlights the advantage of using an ADO II-AS device, which can be delivered via a four French delivery system with no arterial complications.
